# Extraction of Protein and Bioactive Compounds from Mediterranean Red Algae (*Sphaerococcus coronopifolius* and *Gelidium spinosum*) Using Various Innovative Pretreatment Strategies

**DOI:** 10.3390/foods13091362

**Published:** 2024-04-28

**Authors:** Jihen Dhaouafi, Naima Nedjar, Mourad Jridi, Montassar Romdhani, Rafik Balti

**Affiliations:** 1 Laboratory of Functional Physiology and Valorization of Bioresources, Higher Institute of Biotechnology of Beja, University of Jendouba, Avenue Habib Bourguiba, BP, 382, Beja 9000, Tunisia; jihenedhaouefiii@gmail.com (J.D.); jridimourad@gmail.com (M.J.); romdhanimontassar4@gmail.com (M.R.); 2 UMR Transfrontalière BioEcoAgro N°1158, Université Lille, INRAE, Université Liège, UPJV, YNCREA, Université Artois, Université Littoral Côte d’Opale, ICV—Institut Charles Viollette, 59000 Lille, France; naima.nedjar@univ-lille.fr; 3 Université Paris-Saclay, CentraleSupélec, Laboratoire de Génie des Procédés et Matériaux, Centre Européen de Biotechnologie et de Bioéconomie (CEBB), 3 rue des Rouges Terres, 51110 Pomacle, France

**Keywords:** red macroalgae, *Sphaerococcus coronopifolius*, *Gelidium spinosum*, eco-friendly cell disruption methods, protein extraction, antioxidant activity

## Abstract

In this study, the release of proteins and other biomolecules into an aqueous media from two red macroalgae (*Sphaerococcus coronopifolius* and *Gelidium spinosum*) was studied using eight different cell disruption techniques. The contents of carbohydrates, pigments, and phenolic compounds coextracted with proteins were quantified. In addition, morphological changes at the cellular level in response to the different pretreatment methods were observed by an optical microscope. Finally, the antioxidant capacity of obtained protein extracts was evaluated using three *in vitro* tests. For both *S. coronopifolius* and *G. spinosum*, ultrasonication for 60 min proved to be the most effective technique for protein extraction, yielding values of 3.46 ± 0.06 mg/g DW and 9.73 ± 0.41 mg/g DW, respectively. Furthermore, the highest total contents of phenolic compounds, flavonoids, and carbohydrates were also recorded with the same method. However, the highest pigment contents were found with ultrasonication for 15 min. Interestingly, relatively high antioxidant activities like radical scavenging activity (31.57–65.16%), reducing power (0.51–1.70, OD at 700 nm), and ferrous iron-chelating activity (28.76–61.37%) were exerted by the different protein extracts whatever the pretreatment method applied. This antioxidant potency could be attributed to the presence of polyphenolic compounds, pigments, and/or other bioactive substances in these extracts. Among all the used techniques, ultrasonication pretreatment for 60 min appears to be the most efficient method in terms of destroying the macroalgae cell wall and extracting the molecules of interest, especially proteins. The protein fractions derived from the two red macroalgae under these conditions were precipitated with ammonium sulfate, lyophilized, and their molecular weight distribution was determined using SDS-PAGE. Our results showed that the major protein bands were observed between 25 kDa and 60 kDa for *S. coronopifolius* and ranged from 20 kDa to 150 kDa for *G. spinosum*. These findings indicated that ultrasonication for 60 min could be sufficient to disrupt the algae cells for obtaining protein-rich extracts with promising biological properties, especially antioxidant activity.

## 1. Introduction

Already struggling with seven billion humans, the planet’s resources are under enormous and unbearable pressure due to population growth and the increase in food production during the past decades. The global demand for protein is expected to escalate, exacerbating the need for more sustainable production systems to reduce the carbon footprint. These trends encourage the search for alternative protein sources, such as plants, microorganisms, insects, seaweed, etc., to substitute livestock protein-based diets [1]. Particularly, marine macroalgae are significantly rich in protein and also contain a wide range of nutritional and bioactive compounds, such as polysaccharides, pigments, minerals, vitamins, fatty acids, polyphenols, and peptides. These bioactive substances are associated with several health benefits including antioxidant, antibacterial, antihypertensive, antidiabetic, immunomodulatory, anti-inflammatory, and antiviral properties [2,3,4,5].

For many decades, macroalgae have been used as natural ingredients in traditional medicine and cosmetic formulations due to their richness in biologically active components [6]. In addition, macroalgae have long been consumed extensively as fresh human food around the world, especially in Asian countries [7]. Furthermore, macroalgae biomass has been discovered to be a high-quality protein-rich food, making it a sustainable alternative protein source to address current global security challenges [8]. It is well known that red macroalgae have high protein levels, which sometimes exceed conventional protein sources like soybeans, cereals, eggs, and fish [9]. Today, the macroalgae protein market is growing continuously and is projected to reach $1.131 billion by 2027 [10]. Therefore, significant developments are required to efficiently use macroalgae as a sustainable protein supply.

In terms of the profile of proteins and their derivatives, macroalgae contain significant amounts of enzymes, glycoproteins, lectins, peptides, and amino acids, as well as phycobiliproteins, which are the major photosynthetic accessory pigments in cyanobacteria and red algae [11]. The successful generation of bioactive peptides from the enzymatic hydrolysis of macroalgae proteins has been reported. Such peptides can produce a wide range of bioactive effects and can be used as preservatives and functional ingredients to enhance the sensory characteristics of food matrices. In fact, they are considered safer than some synthetic additives, as they present with higher biofunctionality and biospecificity to target cells, and are rarely associated with adverse effects [12]. Recently, seaweed fermentation has demonstrated the potential to generate novel compounds, including bioactive peptides and polysaccharides, processed phenolic compounds, enzymes, and organic acids. This biological process of algal tissues and extracts can be used to create novel food and nutraceutical products with high bioactivity and sensory qualities [13]. Red macroalgal biomass can generate hydrocolloids, proteins, and other valuable unique biomolecules, and they are in high demand in the food, cosmetic, medicine, and pharmaceutical industries [14].

However, the high structural complexity and rigidity of the algal cell wall is a major obstacle to the efficient extraction of intracellular bioactive ingredients, principally proteins and their derivates [15,16]. On the other hand, macroalgae proteins are attached to non-protein components such as polysaccharides (agar, alginates, and carrageenan) and polyphenols [17,18], which is considered among the key factors affecting protein extraction efficiency [19]. Red macroalgae cell walls are composed of a combination of cellulose and cellulose-like polysaccharides, forming the primary barrier to accessing and extracting algal proteins and other intracellular compounds [20].

To extract the internal components of a macroalgae cell, it is necessary to first perform a cell disruption operation that will break down the barrier and allow full access, thus facilitating the release of cellular biomolecules. In recent years, numerous cell disruption and protein extraction techniques have been investigated to enhance the extraction yield and functional properties of macroalgae protein extracts [8]. Hence, several strategies for breaking the cell wall of algae have been evaluated to recover different components, including bead-beating [21,22], ultrasonication [23,24], microwave radiation [25], enzymatic hydrolysis [9,26], cell homogenizing [27], and high-pressure cell disruption [28]. All these extraction methods improve the mass transfer rate and increase the availability of protein and other high value-added components [29]. Until now, there is no proper method to apply to all macroalgae. The extraction approach must be assessed for each species, and the pros and cons must be evaluated regarding the biomass composition to ensure the optimal protocol for obtaining protein-rich fractions.

Importantly, the methodology applied for protein extraction results in the simultaneous release of a wide range of bioactive compounds and therefore significantly affects the chemical composition of the final extract. During the process of protein extraction from *Ulva* sp. and *Gracilaria* sp., valuable phytochemicals such as phenolic compounds were co-extracted, which increases the nutritional value of the final products [30]. In fact, polyphenols are bioactive metabolites characteristic of marine macroalgae, and which are very beneficial to human health, mainly as antioxidant agents [31,32].

The objectives of the present study were first to develop an efficient method for obtaining protein-rich fractions from two red macroalgae *Sphaerococcus coronopifolius* (*Gigartinales, Sphaerococcaceae*) and *Geledium spinosum* (*Gelidiales, Rhodymeniophycidae*). The second objective was to quantify the biomolecules co-extracted concomitantly with proteins and evaluate the antioxidant power of the obtained extracts for possible food and nutraceutical purposes.

## 2. Materials and Methods

### 2.1. Chemicals and Reagents

All chemicals and reagents, including sulfuric acid (H_2_SO_4_), boric acid, hydrochloric acid (HCl), ammonium sulfate (NH_4_)_2_SO_4_, t-butanol, tris-HCl buffer, Bradford reagent, bovine serum albumin, glucose, Laemmli buffer, glycine, sodium dodecyl sulfate (SDS), phenol reagent, acetone, gallic acid, Folin–Ciocalteu reagent, sodium carbonate (Na_2_CO_3_), sodium nitrite (NaNO_2_), sodium hydroxide (NaOH), aluminum chloride (AlCl_3_), quercetin, ethanol, 1,1-diphenyl-2-picrylhydrazyl (DPPH), 3,4-dihydro-6-hydroxy-2,5,7,8-tetramethyl-2H-1-benzopyran-2-carboxylic acid (Trolox), butylated hydroxyanisole (BHA), ferric chloride (FeCl_2_), ferrozine potassium ferricyanide, trichloroacetic acid (TCA), and 3.5 kDa MWCO dialysis tubing, were obtained from Sigma-Aldrich (Saint-Quentin-Fallavier, France).

### 2.2. Collection and Preparation of Algal Materials

The *S. coronopifolius* species was collected in January and February 2019 from Menzel Abderrahmane city, Bizerte, located in the North-West of Tunisia (37°13′48″ N and 9°51′36″ E). Similarly, the *G. spinosum* species was collected in the same period from the Monastir region, which is in the East of Tunisia (35°46′ N and 10°49′ E). The collected samples were packed in polyethylene bags and transported to the laboratory within 2 h. Upon arrival, the red macroalgal biomass was rinsed with tap water to remove epiphytes, sediment, and potential contaminants. Fresh macroalgae were subsequently dried in a dark room at ambient temperature for a few weeks. The dried samples were then ground to a fine powder using a blender (Knife Mill Grindomix GM 200, Retsch, Haan, Germany) and kept in airtight glass jars.

### 2.3. Determination of Crude Protein

The organic nitrogen content was quantified using the Kjeldahl procedure [33]. A total of 0.5 g of dried macroalgae was digested in a Kjeldaltherm^®^ block digestion unit (Gerhardt, Königswinter, Germany) in 15 mL of concentrated H_2_SO_4_ and one tablet (2.5 g) of Kjeldahl catalyst for 1 h. Digestion was completed on the production of a clear solution. Steam distillation after Kjeldahl digestion was carried out in a Vapodest^®^ 33 unit (Gerhardt, Germany). The distillate was collected in an Erlenmeyer flask containing 15 mL of 4% (*v*/*v*) aqueous boric acid solution brought to a final volume of 50 mL. The titration was performed manually using a standard HCl solution (0.1 M). Nitrogen content, given in g of nitrogen per 100 g of the sample, was calculated using the following numerical equation (Equation (1)):

(1) $$Nitrogen\;content(\%)=1.4007\times \frac{\left(V_{sample}\;\left(\mathrm{mL}\right)-V_{blank}\;\left(\mathrm{mL}\right)\right)\times 0.1}{Weight\;of\;sample\;(g)}$$

where V_blank_ and V_sample_ are the volumes of hydrochloric acid consumed during the titration of the reagent blank and sample, respectively. A factor of 6.25 was used to convert the nitrogen value to protein [34]. All measurements were performed in triplicate.

### 2.4. Pretreatment Methods for Red Macroalgae Cell Disruption and Protein Extraction

**Figure 1:**
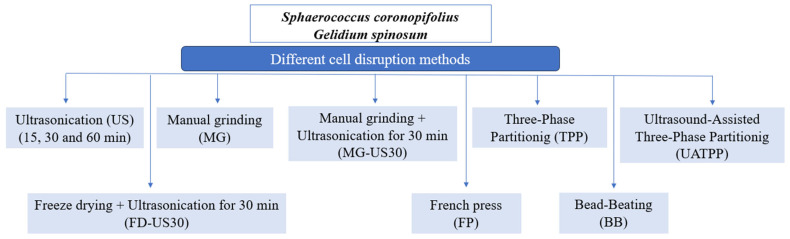
A schematic of different cell disruption and protein extraction methods applied to two red macroalgae (*S. coronopifolius* and *G. spinosum*).

The different pretreatment techniques applied to red macroalgae biomasses to disrupt the cell wall and protein extraction are presented in Figure 1. All pretreatments were carried out in triplicate.

#### 2.4.1. Control

One gram of red seaweed powder was dispersed in 100 mL of distilled water for 2 h, and the supernatant was then recovered by centrifugation at 6000× *g* for 20 min at 4 °C. This preparation served as a control to compare with the other pretreatment methods.

#### 2.4.2. Ultrasonication (US)

Red macroalgae samples were subjected to ultrasonication pretreatment using an ultrasonic cell disruptor (Omni Sonic Ruptor 4000, Kennesaw, GA, USA), following the methodology of Safi et al. [35] with slight modifications as described by Malik et al. [36]. One gram of the macroalgae powder was dissolved in 100 mL of distilled water and then ultrasonically treated at a frequency of 20 kHz and a power of 200 W with amplitudes set at 90% (with a pulse duration of on-time 15 s and off-time 15 s). The soluble fractions were collected by centrifugation at 6000× *g* for 20 min at 4 °C.

#### 2.4.3. Manual Grinding (MG)

Dry macroalgae were manually ground using a mortar for 10 min, and then 1 g was dispersed in 100 mL of distilled water for 2 h. The supernatant was recovered by centrifugation at 6000× *g* for 20 min at 4 °C.

#### 2.4.4. Combination of Manual Grinding and Ultrasonication for 30 min (MG-US30)

The red macroalgae powder samples were manually ground separately using a mortar for 10 min, and then 1 g of each sample was dissolved in 100 mL of distilled water. The samples were placed in a beaker before ultrasound pretreatment. The pretreatment was performed at a frequency of 20 kHz and a power output of 200 W (amplitudes of 90%) for 30 min (pulse duration of on-time 15 s and off-time 15 s). The mixtures were centrifuged at 6000× *g* for 20 min at 4 °C and the supernatants were collected for biochemical analyses.

#### 2.4.5. Three-Phase Partitioning (TPP)

The TPP technique was carried out in accordance with the previously published method of Chia et al. [37] with minor modifications. Before combining with salt solution, 1 wt% of red macroalgae biomass was first dissolved in deionized water. Then, 5 mL of pure t-butanol and 5 mL of 30% saturation (NH_4_)_2_SO_4_ were added. The mixture was agitated using a magnetic stirrer at 200 rpm for 1 h and was allowed to separate for 30 min at room temperature. The three phases were observed and separated carefully by pipetting them out from the beaker. The intermediate protein precipitate was dissolved in an appropriate amount of tris-HCl buffer and analyzed for soluble protein and other biomolecule contents.

#### 2.4.6. Ultrasound-Assisted Three-Phase Partitioning (UATPP)

The UATPP procedure was carried out using an ultrasonic cell disruptor (Omni Sonic Ruptor 4000, Kennesaw, GA, USA). For the comparison study, the initial parameters of UATPP such as the working volume, saturation of salt solution, and weight of biomass were similar to the TPP method. The preparation and mixing procedure of UATPP was similar to TPP [37]. The ultrasonic treatment of the mixture was operated at 20 kHz and a power output of 200 W with an amplitude of 90% for 30 min (pulse duration of on-time 15 s and off-time 15 s). The treated solution was then taken out and allowed to separate for 30 min at room temperature.

#### 2.4.7. Combination of Freeze Drying with Ultrasonication for 30 Min (FD-US30)

Frozen macroalgae pastes (10 g) were directly introduced to a Lyovapor™ L-200 freeze dryer (BÜCHI Labortechnik AG, Flawil, Switzerland). The pressure was reduced to 0.0010 bar and the temperature was further decreased to −80 °C and freeze-drying was conducted under vacuum for 48 h. Then, one gram of each freeze-dried powder was dissolved in 100 mL of distilled water to ensure optimal sample homogeneity. Finally, the ultrasound treatment was carried out at 20 kHz for 30 min with a power output of 200 W (pulse duration of on-time 15 s and off-time 15 s), followed by centrifugation at 6000× *g* for 20 min at 4 °C for the recovery of the soluble phase.

#### 2.4.8. French Press (FP)

The dried macroalgae biomasses were dispersed in distilled water at 10 g/L and vigorously mixed in a vortex (Vortex 3, IKA, Staufen, Germany) to ensure the homogeneity of the macroalgal samples. A high-pressure homogenization method using a One-Shot Cell Disrupter (Constant Systems Ltd., Warwickshire, UK) was applied to the red macroalgae suspension in one pass at a pressure of 2700 bar [35]. The working volume in this study was fixed at 8 mL. In this process, the macroalgal cells are forced to flow through a very small orifice under high-pressure conditions, and, as a result, they could be disrupted by synergistic mechanical effects, such as cavitation, turbulence, and shear stress [38]. Water-soluble protein extracts were obtained by centrifugation (6000× *g*, 20 min, 4 °C).

#### 2.4.9. Bead-Beating (BB)

Red macroalgae cells were disrupted with the bead-beating method according to Suarez Garcia et al. [39] with minor modifications. Macroalgae aqueous suspensions (10 g/L) were transferred to MN Bead Tubes Type C containing 1–3 mm corundum beads (Macherey-Nagel, Düren, Germany). The samples were subjected to intense mixing using a Fastprep-24 5G^TM^ bead beater (MP Biomedicals, Santa Ana, CA, USA) for three cycles at 6 m/s for 60 s each. A cooling phase of 2 min in between cycles was fixed to avoid overheating and the decomposition of metabolites (total extraction time ≈ 10 min, T = 25 °C ± 2 °C).

#### 2.4.10. Mass Extraction Yield Calculation

All extracts obtained were freeze-dried and weighed. In order to assess the extraction performances of the evaluated cell disruption methods, the mass extraction yield was calculated and used as an indicator of the effectiveness of biomass pretreatments. Mass extraction yields (Y) were calculated according to Equation (2):

(2) $$Y\;(\%)=\frac{weight\;of\;freeze-dried\;extract\;(g)}{weight\;of\;red\;macroalgae\;powder\;(g)}\times 100$$

All calculations are conducted on a dry-weight (DW) basis.

### 2.5. Optical Microscopic Observation

Suspensions of the untreated and treated red macroalgae were analyzed with an Optika B-190TB light microscope (OPTIKA, Ponteranica, Italy). Digital images were taken with a 3.1-megapixel digital color microphotography camera C-B3A (OPTIKA, Ponteranica, Italy). Acquired images were analyzed and processed using the OPTIKA vision lite 2.1 Software (OPTIKA, Ponteranica, Italy).

### 2.6. Estimation of Total Soluble Protein

The soluble protein concentration was determined using the dye-binding Bradford assay [40]. Briefly, 0.25 mL of the sample was mixed with 2.5 mL of Bradford reagent (1:50 *v*/*v*) and incubated at room temperature for 15 min. The absorbance of the samples was then measured at 595 nm using a UV–VIS spectrophotometer (SpectraMax^®^ ABS Plus, San Jose, CA, USA) and the soluble protein concentration was calculated using the standard calibration of bovine serum albumin. The measurements were performed in triplicate.

### 2.7. Protein Profile by SDS-PAGE

Protein-denaturing sodium dodecyl sulfate polyacrylamide gel electrophoresis (SDS-PAGE) of each extract was performed according to the Laemmli method [41]. Briefly, the extracted proteins were solubilized in ultrapure water at a concentration of 2 mg/mL and diluted in Laemmli buffer containing β-mercaptoethanol and SDS before heating at 95 °C for 10 min. Then, 25 μL samples and 4 μL molecular mass marker solutions (Precision Plus Protein^TM^ Standards, Bio-Rad, Marnes-la-Coquette, France) were deposited on an Any-kD^TM^ Mini-Protean^®^ TGX Stain-free^TM^ gel (Bio-Rad). Protein migration was allowed to occur for 1 h in a buffer containing Tris-base (25 mM), glycine (0.19 mM), and SDS (3.5 mM) under a constant voltage of 120 V. Thereafter, fluorescence generated by the reaction between the gel-trihalo compounds and tryptophan residues of the proteins was revealed after 5 min of activation time with the Gel Doc^TM^ XR+ system and Image Lab 6.1.0 software (Bio-Rad).

### 2.8. Quantification of Co-Extracted Compounds

Following the different pretreatment methods applied, other intracellular molecules derived from red macroalgae, such as polyphenols, carbohydrates, and pigments, could be released simultaneously with the proteins. Indeed, the quantification of the content of these co-extracted compounds in the final protein extract was carried out.

#### 2.8.1. Total Carbohydrate Analysis

Total carbohydrate content was evaluated by the colorimetric method after adding phenol and sulfuric acid as described by Dubois et al. [42]. Typically, 500 μL of the sample was introduced at the bottom of a 15 mL polypropylene falcon tube. Then, 500 μL of phenol solution (50 g/L) was added to 2.5 mL of sulfuric acid (>96%). After 30 min of incubation at ambient temperature, the absorbance at 485 nm was measured using a UV–VIS spectrophotometer. The calibration curve was made using D-glucose and each sample was analyzed in triplicate.

#### 2.8.2. Pigment Analysis

The quantitative estimation of chlorophyll a and chlorophyll b was carried out with the method of Arnon [43], while carotenoids were determined by following Kirk and Allen [44]. Acetone (80%) was used as the extractant solvent and the absorbance of the extracted solution was measured using a UV–VIS spectrophotometer at the wavelengths of 480, 645, and 663 nm. The chlorophyll and carotenoid contents were calculated using the following formulas (Equations (3)–(5)) in triplicate for each pretreatment and expressed as mg/g of DW.

(3) $$\;Chlorophyll\;a\;(\mathrm{mg}/g\;\mathrm{DW})=\frac{\left(12.7-{\;A}_{663}\right)-\left(2.69\;\times {\;A}_{645}\right)\times Final\;volume\;of\;extract\;(\mathrm{mL})\;}{Weight\;of\;pretreated\;macroalgae\;(g)}$$

(4) $$\;Chlorophyll\;b\;(\mathrm{mg}/g\;\mathrm{DW})=\frac{\left(22.9-{\;A}_{645}\right)-\left(4.86\times {\;A}_{663}\right)\times Final\;volume\;of\;extract\;(\mathrm{mL})\;}{Weight\;of\;pretreated\;macroalgae\;(g)}$$

(5) $$Carotenoid\;(\mathrm{mg}/g\;\mathrm{DW})=\frac{4\times A_{480}\times Final\;volume\;of\;extract\;(\mathrm{mL})}{Weight\;of\;pretreated\;macroalgae\;(g)}$$

where A_480_, A_645_, and A_663_ are the absorbances at wavelengths of 480, 645, and 663 nm, respectively. Each sample was analyzed in triplicate.

#### 2.8.3. Quantitative Polyphenol Analysis

Total phenolic content (TPC) was measured with a colorimetric assay using the Folin–Ciocalteu phenol reagent [45] and using gallic acid as the standard phenolic compound. Briefly, 50 µL of the sample was added to 120 µL of Folin–Ciocalteu reagent and 2 mL of distilled water and mixed thoroughly for 5 min. Then, 375 µL of 10% (*w*/*v*) sodium carbonate was added and the mixture was allowed to stand for 2 h at room temperature. The absorbance was measured at 765 nm using a UV–VIS spectrophotometer. TPC was expressed as mg gallic acid equivalents (GAE)/g DW. The test was carried out in triplicate.

Total flavonoid content (TFC) was determined using the aluminum chloride colorimetric method [46] and using quercetin as the condensed flavonoid standard. Briefly, 400 µL of the sample was mixed with 120 µL of 5% (*w*/*v*) NaNO_2_ and 120 µL of 10% (*w*/*v*) AlCl_3_ was added. After 6 min, 800 µL of NaOH (1 M) was added and the absorbance of the mixture was measured at 510 nm. TFC was expressed as mg quercetin equivalents (QE)/g DW. The test was carried out in triplicate.

### 2.9. Antioxidant Assays

The antioxidant activity of obtained extracts was determined by different *in vitro* methods, such as the DPPH free radical scavenging, ferrous ion-chelating ability, and reducing power assays. All tests were carried out in triplicate and average values were considered.

#### 2.9.1. DPPH Free Radical Scavenging Assay

DPPH free radical scavenging activity was measured using the method described by Bersuder et al. [47]. Briefly, a 500 µL test sample was mixed with 375 µL of 99.5% ethanol and 125 µL of 0.02 mM DPPH ethanol solution. This mixture was shaken then kept in the dark at room temperature for 30 min before measuring its absorbance at 517 nm. DPPH radical scavenging activity was calculated according to the following equation (Equation (6)):

(6) $$DPPH\;radical\text{-}scavenging\;activity\;\left(\%\right)=(1-\frac{A_{517}\;of\;sample}{A_{517}\;of\;control})\times 100$$

The control was conducted in the same manner, except that distilled water was used instead of the sample. Trolox (6-hydroxy-2,5,7,8-tetramethylchroman-2-carboxylic acid) at 1 mg/mL was used as the standard.

#### 2.9.2. Ferrous Ion-Chelating Ability Assay

The chelating activity on Fe^2+^ was determined using the method described by Decker and Welch [48]. An aliquot of 100 µL of the sample solution was mixed with 50 µL of 2 mM FeCl_2_ and 450 µL of distilled water. The mixture was then reacted with 200 µL of 5 mM ferrozine for 10 min at room temperature. The absorbance of the Fe^2+^–ferrozine complex with red or violet color was read at 562 nm. The control was prepared in the same manner except that distilled water was used instead of the sample. Butylated hydroxyanisole (BHA) tested at 1 mg/mL was used as a reference. Chelating activity was then calculated as follows (Equation (7)):

(7) $$Chelating\;activity\;(\%)=(1-\frac{A_{562}\;of\;sample}{A_{562}\;of\;control})\times 100$$

#### 2.9.3. Reducing Power Assay

The ability of the samples to reduce iron (III) was determined according to the method of Yildirim et al. [49] with slight modifications. An aliquot of 0.5 mL of each sample was mixed with 1.25 mL of 0.2 M phosphate buffer (pH 6.6) and 1.25 mL of 1% potassium ferricyanide. The mixture was incubated at 50 °C for 30 min, followed by the addition of 1.25 mL of 10% (*w*/*v*) trichloroacetic acid. The mixture was then centrifuged at 3000× *g* for 10 min. Finally, 1.25 mL of the supernatant was mixed with 1.25 mL of distilled water and 0.25 mL of 0.1% (*w*/*v*) ferric chloride. After a 10 min reaction, the absorbance of the resulting solution was measured at 700 nm. Increased absorbance of the reaction mixture indicated increased reducing power. BHA (1 mg/mL) was used as a positive control.

### 2.10. Statistical Analysis

Each extract was independently produced in triplicate. All the experimental tests were performed at least in triplicate. Values are expressed as mean ± standard deviation (SD). Data analysis was carried out with the GraphPad Prism 9.0 software (GraphPad Software, Inc., San Diego, CA, USA) using a one-way ANOVA analysis followed by post hoc Tukey’s honestly significant difference (HSD) tests. Differences were considered statistically significant at *p* < 0.05.

## 3. Results and Discussion

### 3.1. Total Protein Contents of S. coronopifolius and G. spinosum

The total protein contents of the raw materials were determined using the Kjeldahl method by converting elemental total nitrogen into protein percentage. The protein content of *S. coronopifolius* was 17.62 ± 0.20%. Consistent findings were reported by Patarra et al. [50] for the same species, recording a protein content of 19.56%. On the other hand, *G. spinosum* exhibited a protein content of 22.71 ± 0.31%. This value appeared slightly lower compared to the results presented by Ben Said et al. [51], who reported a protein value of 29% for *G. spinosum* harvested from the Monastir coasts (Tunisia). Generally, the content of proteins from macroalgae is strongly influenced by the geographical origin, species, and season [52,53]. In fact, higher levels of proteins are reported in winter [54]. For example, the protein content of *G. spinosum* varied between 18 and 29% in April and January, respectively [51]. Also, our results are comparable to those reported for other red macroalgae species such as *Gracilaria edulis* (25.3%) [55], *Palmaria palmata* (15.3%) [56], *Chondracanthus chamissoi* (17.6%) [57], and *Pyropia orbicularis* (13.6%) [58].

Despite the high nutritional value of algal proteins [9], their availability is limited by the rigidity of the algal membrane. For this reason, our study aimed to evaluate the proteins released in the aqueous media after different cell disruptions. Compounds such as polyphenolic compounds, carbohydrates, and pigments are extracted simultaneously with the proteins during the different treatments. The results are based not only on the mechanical rigidity of each macroalga cell wall but also on its chemical properties.

### 3.2. Optical Scanning Microscopy Morphology Observation of Macroalgae Cells

**Figure 2:**
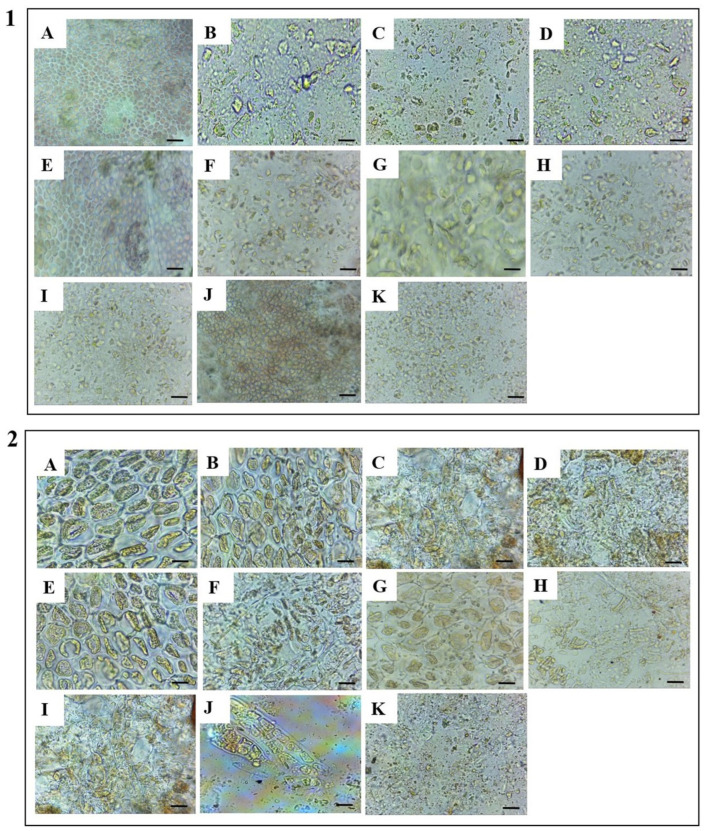
Cell morphology observation of *S. coronopifolius* (**1**) and *G. spinosum* (**2**) under 40× magnification: untreated cells (**A**), US for 15 min (**B**), US for 30 min (**C**), US for 60 min (**D**), MG (**E**), MG-US30 (**F**), TPP (**G**), UATPP (**H**), FD-US30 (**I**), BB (**J**), and FP (**K**). Scale bar, 10 mm.

To better interpret the efficacy of each pretreatment, morphological changes of the *S. coronopifolius* and *G. spinosum* control and pretreated cells were observed using an optical microscope. The results are displayed in Figure 2.

Figure 2(1A,2A) shows the image of algal cells of *S. coronopifolius* and *G. spinosum*, respectively, before being subjected to sonication (untreated cells). Regarding *S. coronopifolius*, the cells are spherical and have intact intracellular compartments (2.00–3.5 mm in diameter). In contrast, the untreated cells of *G. spinosum* have an ellipsoidal shape, with the intracellular compartments intact within the cell (4.00–10.00 mm in diameter).

Figure 2(1B–K,2B–K) shows the morphology of the cells after different pretreatments, where the cells were subjected to the cell disintegration treatments. As can be observed from Figure 2(1B,C,2B,C), the cells treated with US for 30 and 15 min were not disrupted significantly. As shown in Figure 2(1D,2D), the cells were fragmented after US for 60 min. This proves that sonication is important for breaking the cell wall to facilitate the release of protein and other compounds. Acoustic cavitation can effectively destroy the gas vacuoles that control the floating of algal cells in water [59,60]. Therefore, sonication should reduce the suspension of algal cells and accelerate their sedimentation.

On the other hand, when pretreated with FD-US30 (Figure 2(1I) and Figure 2(2I) for *S. coronopifolius* and *G. spinosum*, respectively), the majority of the cells were broken, while some of them remained intact and cells maintained their globular form. Combination treatment, such as MG-US30, showed that the cells are similar to the cells observed from US30 only (Figure 2(1F) and Figure 2(2F) for *S. coronopifolius* and *G. spinosum*, respectively).

In the case of BB treatment (Figure 2(1J) and Figure 2(2J) for *S. coronopifolius* and *G. spinosum*, respectively), the cells were partially disrupted with the presence of some organelles that were liberated, and many cells were quiet in this pretreatment. High-pressure cell disruption using FP (Figure 2(1K) and Figure 2(2K) for *S. coronopifolius* and *G. spinosum*, respectively) was relatively efficient for the two red macroalgae; the majority of cells were broken while some of them remained intact.

### 3.3. Mass Extraction Yield

**Figure 3:**
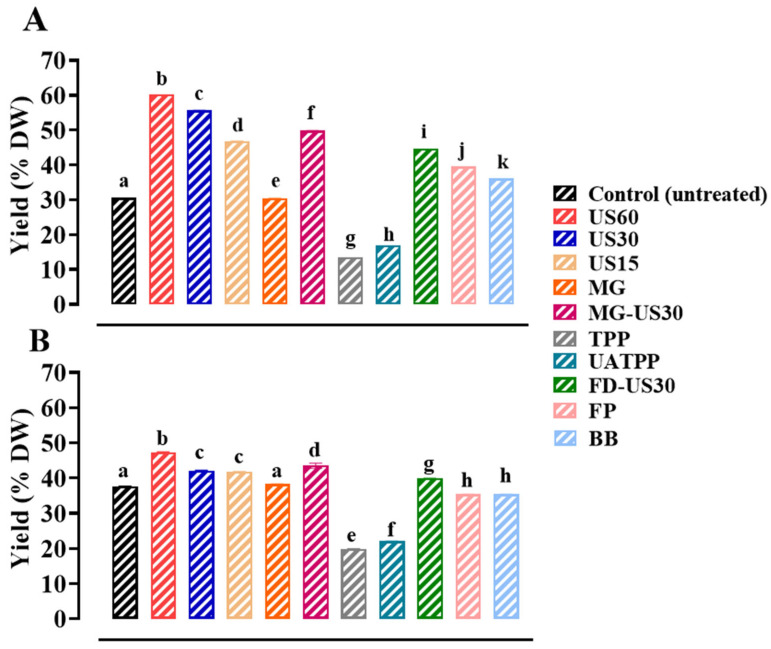
Mass extraction yields of *S. coronopifolius* (**A**) and *G. spinosum* (**B**) pretreated with different cell disruption techniques. a, b, c, d, e, f, g, h and k: different letters mean significant differences between extracts (*p* < 0.05). Results are expressed as average ± standard deviation (SD) (*n* = 3). US, ultrasonication; MG, manual grinding; TPP, three-phase partitioning; UATPP, ultrasonication-assisted three-phase partitioning; MG-US30, manual grinding + ultrasonication for 30 min; FD-US30, freeze drying + ultrasonication for 30 min; FP, French press; and BB, bead-beating.

The mass recovery yields achieved through various disruption methods are depicted in Figure 3. The mass extraction yields ranged from 13.47 ± 0.003% DW to 60.19 ± 0.007% DW and from 19.86 ± 0.07% DW to 47.43 ± 0.14% DW for *S. coronopifolius* and *G. spinosum,* respectively. Among all tested techniques, the US 60 min cell disruption appears to be the best technique (*p* < 0.05) for both *S. coronopifolius* (60.19% DW) and *G. spinosum* (47.44% DW) compared to the untreated cells (30.53 ± 0.07% DW and 37.68 ± 0.07% for *S. coronopifolius* and *G. spinosum*, respectively). These findings showed the necessity of effective cell disruption to maximize the release of intracellular compounds from the two red macroalgae.

US power demonstrated a significant effect (*p* < 0.05) on the increase of the extraction yields for *S. coronopifolius* and *G. spinosum* (Figure 3), unlike those obtained without ultrasound pretreatment (0 W/L) such as in the untreated cells, TPP, and UATPP. This indicated the positive effect of the ultrasonication method on the extraction of intracellular compounds. Sonication duration is also a very important parameter that determines the treatment efficiency. By applying a longer extraction time using US for 60 min, the macroalgae biomasses are subjected to prolonged exposure to ultrasonic waves which generate an implosion of cavitation bubbles in the fluid. These conditions are easily capable of disrupting cell walls and membranes and releasing intracellular compounds in an efficacious and rapid manner. Intracellular compounds such as proteins, polysaccharides, lipids, vitamins, minerals, and antioxidants can thereby be effectively extracted using power ultrasonics. In view of these high yields, US appears to be a suitable primary extraction method for the disintegration of cell walls, thereby facilitating the release of target compounds from red macroalgae.

Many researchers have been interested in ultrasonication extraction since it provides a greater biomolecule extraction yield [61,62]. The extraction yield of bioactive chemicals (laminarin, fucose, uronic acid, and phenolics) from the brown algae *A. nodosum* is improved by ultrasonication-assisted extraction [26,63]. Previous research has shown that molecular vibrations generated by high-power ultrasonic waves can help disrupt chemical interactions between molecules and facilitate molecular mobility [64]. Usually, the composition of the cell wall must also be considered since it constitutes a determining factor that can significantly influence the efficiency of the extraction [65].

The combined treatment with FD-US30 showed a significantly (*p* < 0.05) lower yield (44.69% DW and 39.88% DW for *S. coronopifolius* and *G. spinosum*, respectively) than the US 30 min alone (55.65% DW and 42.14% DW for *S. coronopifolius* and *G. spinosum*, respectively). It has been demonstrated that the freeze-drying process, which preserves biological materials well, makes protein extraction more difficult for particular algae species [66]. Furthermore, after freeze-drying, the cells become more aggregated, reducing the contact surface with the extracting solvent and potentially affecting the cell wall integrity [67,68]. The obtained results were in line with those of Barbino et al. [69] for the two macroalgae *Sargassum vulgare* and *Chnoospora minima*.

The MG-US30 combined pretreatment is gaining importance due to it resulting in higher yields than US 30 min alone (*p* < 0.05) (49.84% DW and 43.54% DW for *S. coronopifolius* and *G. spinosum,* respectively). For FP pretreatment, the yield was in the order of 39.53% DW and 45.79% DW for *S. coronopifolius* and *G. spinosum,* respectively. On the other hand, the lowest yields were obtained for the TPP and UATPP methods (13.47% DW and 16.89% DW for *S. coronopifolius* and 19.86% DW and 22.07% DW for *G. spinosum*, respectively) due to the rigidity of the cell walls of the studied macroalgae species. Aqueous maceration alone has been shown to generally have a relatively low extraction yield compared to other alternative liquid extraction systems [37].

### 3.4. Release of Soluble Protein

**Figure 4:**
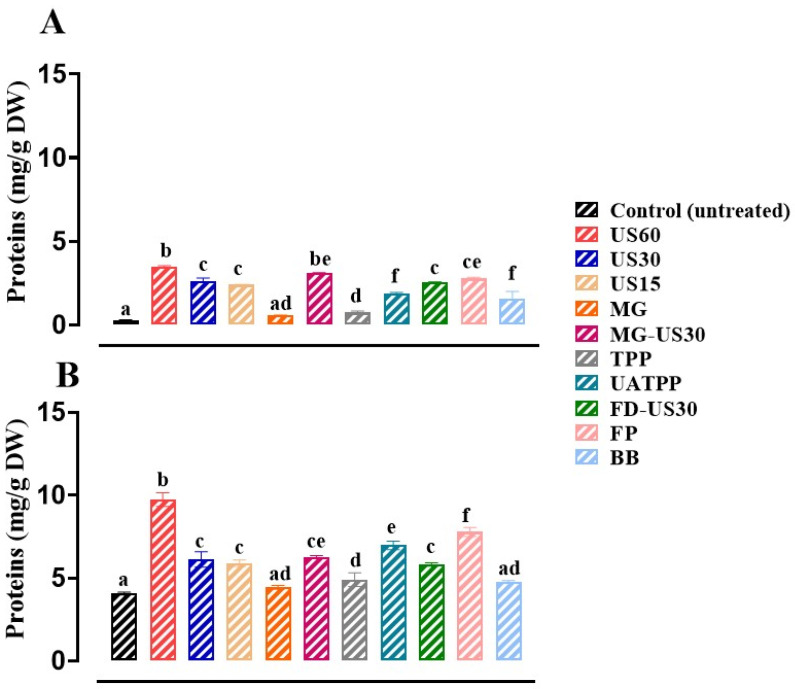
Protein contents of *S. coronopifolius* (**A**) and *G. spinosum* (**B**) extracts using different techniques of cell disruption. a, b, c, d, e, and f: different letters mean significant differences between extracts (*p* < 0.05). Results are expressed as average ± standard deviation (SD) (*n* = 3). US, ultrasonication; MG, manual grinding; TPP, three-phase partitioning; UATPP, ultrasonication-assisted three-phase partitioning; MG-US30, manual grinding + ultrasonication for 30 min; FD-US30, freeze drying + ultrasonication for 30 min; FP, French press; and BB, bead-beating.

Protein extraction yields from algae sources are significantly higher than those from protein-rich crops such as lupin, soybean, and legumes [70]. Therefore, significant developments are required to efficiently exploit marine macroalgae as an alternative sustainable protein source. This study compared the efficiency of different protein extraction techniques from *S. coronopifolius* and *G. spinosum*. Figure 4 shows the protein contents obtained by the different pretreatment methods of biomass and untreated cells. The extracted protein content was very low in untreated cells for *S. coronopifolius* (0.24 mg/g DW) and it is in the order of 4.05 mg/g DW for *G. spinosum*, which confirms the need for efficient cell disruption to enhance protein release. These results were in line with expectations as the extraction was carried out in water. However, the osmosis phenomenon was not strongly effective for red macroalgae, which are known to have rigid cell walls [37].

For both macroalgae, the protein levels after 10 min of MG have no significant difference (*p* > 0.05) compared to untreated cells. On the other hand, the comparison of other techniques revealed the superiority of US 60 min, followed by the FP method. The total quantity of extractable protein from *S. coronopifolius* was 3.43 ± 0.06 mg/g DW. Similarly, for the red macroalga *G. spinosum*, the protein amount was 9.73 ± 0.41 mg/g DW when US was applied for 60 min. This was further supported by microscopic observation, which revealed a complete structural alteration (Figure 2).

Considering previous data on the time required for effective ultrasound pretreatment [71,72], the evaluation of the US technique was carried out with different pretreatment periods ranging from 15 to 60 min. Our study demonstrated that US for 60 min coupled with a power output of 200 W significantly affects the concentrations of protein released. Similar results were reported by Al-Zuhair et al. [73], illustrating how ultrasound influenced the levels of extracted protein from various microalgae species including *Chlorella* sp., *Ankistrodesmus braunii*, *Pseudochlorococcum* sp., *Tetraselmis* sp., and *Nannochloropsis* sp. Furthermore, our results are consistent with the findings of Pernet and Tremblay [74] who determined that different disruption methods, particularly sonication techniques, significantly affected the extracted protein levels from the marine-centric diatom, *Chaetoceros gracilis.* In addition, FP application (600 MPa for 4 min) to facilitate protein extraction from two red algae (*Palmaria palmata* and *Chondrus crispus*) was studied by O’ Connor et al. [28]. Indeed, FP treatment at 400 MPa for 20 min of *Soleria chordalis* only resulted in an increase of 2.60% (*w*/*w*) in protein yield [75].

The combined treatment of MG-US30 (3.09 ± 0.03 mg/g DW and 5.19 ± 0.18 mg/g DW for *S. coronopifolius* and *G. spinosum*, respectively) also seems interesting due to the higher protein concentrations compared to the US for 30 min alone (2.61 ± 0.19 mg/g DW and 6.71 ± 0.96 mg/g DW for *S. coronopifolius* and *G. spinosum*, respectively). In addition, the protein contents recorded with UATPP (1.89 ± 0.04 mg/g DW and 6.96 ± 0.25 mg/g DW for *S. coronopifolius* and *G. spinosum*, respectively) are higher (*p* < 0.05) than that obtained with TPP (0.72 ± 0.09 mg/g DW and 4.89 ± 0.41 mg/g DW for *S. coronopifolius* and *G. spinosum*, respectively). The combination with the ultrasonic treatment, capable of cracking the cell wall of macroalgae, probably explains the differences in protein contents between UATPP and TPP. These findings are consistent with those of Chia et al. [37] who reported that UATPP was found to be an improved technique compared to TPP for the extraction of proteins from *Chlorella vulgaris* FSP-E. Moreover, the lowest protein concentrations for both macroalgae were obtained in untreated cells and through MG, especially for *S. coronopifolius*.

The ultrasonication method is based on liquid shear forces caused by high-frequency wave sounds (up to 15–20 kHz). These sound waves form gas bubbles or cavities in the liquid, which reach a threshold size after a certain number of cycles, collapsing and releasing significant amounts of energy. Acoustic cavitation also causes cell wall destruction by increasing local temperatures and producing hydroxyl radicals [76]. It has been reported that ultrasonication with lower frequencies and higher power causes more violent cavitation reactions [77,78,79] and has substantial mechanical effects on solid particles, potentially enhancing mass transfer during extraction [80]. The effect of ultrasound is due to bubble cavitation, which facilitates the disruption of biological matrices [29].

### 3.5. Protein Molecular Weights Profile

US for 1 h appears to be the most effective method for extracting the maximum protein content from both macroalgae. These proteins were precipitated with ammonium sulfate. Recently, ammonium sulfate (80% *w*/*v*) precipitation in combination with dialysis using a 3.5 kDa MWCO membrane of proteins extracted from macroalgae by sonication (1 h at 42 Hz) has been demonstrated [28]. Ammonium sulfate is the salt of choice due to its food-grade status, cost effectiveness, exceptional solubility, and its ability to efficiently stabilize protein structures [81,82].

**Figure 5:**
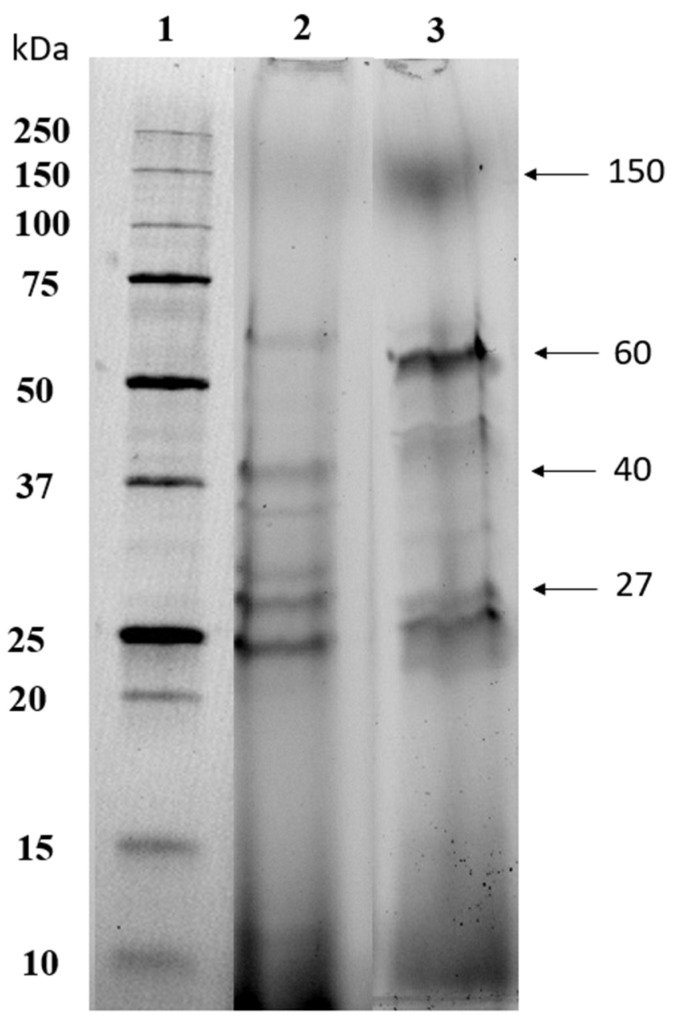
Sodium dodecyl sulphate polyacrylamide gel electrophoresis profiles of red macroalgae soluble protein extracts. Lane 1: molecular weight standards. Lane 2: water soluble protein from *S. coronopifolius*. Lane 3: water soluble protein from *G. spinosum*.

The molecular weight distribution of the extracted macroalgae soluble proteins by US 60 min from *S. coronopifolius* and *G. spinosum* was estimated under denaturing conditions by SDS-PAGE (Figure 5). In general, the SDS-PAGE profile of protein extracts for the two red macroalgae samples showed that the protein bands were resolved clearly without having too much smearing. Some variations in the protein pattern between the two species of red macroalgae were also observed. Effectively, the major protein bands of *S. coronopifolius* were observed between 25 and 60 kDa (Figure 5, lane 2). In contrast, the *G. spinosum* protein extract revealed that a majority showed a pattern containing approximately six discrete bands with molecular weights of 25 to 150 kDa (Figure 5, lane 3). Bands containing abundant proteins were observed in the two species, but their intensity varied among the extracts of red macroalgae. Previous research showed that most protein fractions of red macroalgae visualized by SDS-PAGE ranged between 6.5 and 116 kDa. In addition, band sizes with low molecular weight (<15 kDa), however, varied, revealing the differences in types of proteins among various seaweed samples.

The SDS-PAGE profile of aqueous soluble proteins extracted from milled oven-dried *Palmaria palmata* has presented a greater number of protein bands ranging in size from 15.5 to 97 kDa with four main protein bands ranging from 14.8 to 55 kDa compared to the alkaline soluble protein extract [83].

Phycoerythrin, which is a photosensitive red pigment from the phycobiliprotein family predominantly present in red algae, is composed of three subunits (α, β, and γ) with apparent molecular weights of 18, 20, and 30–33 kDa, respectively. This water-soluble chromoprotein is a large oligomer characterized by the aggregation of its subunits to form a basic unit with different arrangements like the complexes of αβ that can have a molecular weight of about 38 kDa.

### 3.6. Release of Soluble Carbohydrates

**Figure 6:**
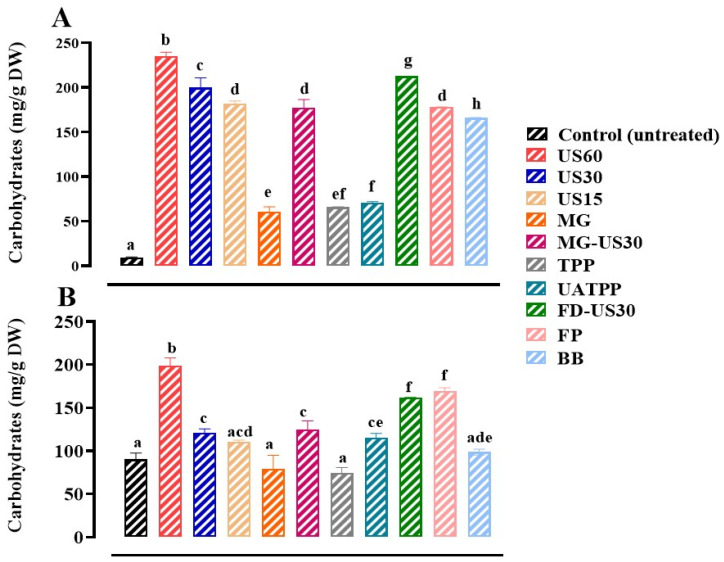
Carbohydrate contents of *S. coronopifolius* (**A**) and *G. spinosum* (**B**) extracts using different techniques of cell disruption. a, b, c, d, e, f, g, and h: different letters mean significant differences between extracts (*p* < 0.05). Results are expressed as average ± standard deviation (SD) (*n* = 3). US, ultrasonication; MG, manual grinding; TPP, three-phase partitioning; UATPP, ultrasonication-assisted three-phase partitioning; MG-US30, manual grinding + ultrasonication for 30 min; FD-US30, freeze drying + ultrasonication for 30 min; FP, French press; and BB, bead-beating.

Separating carbohydrates from proteins proves challenging due to the substantial presence of these polymers within the algal matrix, potentially leading to protein–polysaccharide interactions. Overcoming this challenge still remains a major issue in the extraction of proteins from algae [84]. In the case of *S. coronopifolius*, carbohydrate contents ranged from 9.03 mg/g DW to 235.25 mg/g DW (Figure 6A). Similarly, *G. spinosum* extracts presented values between 79.56 ± 1.56 mg/g DW and 199.18 ± 7.53 mg/g DW (Figure 6B). Notably, the use of US improved the extraction process, yielding significantly (*p* < 0.05) higher carbohydrate contents for both red macroalgae. Moreover, the duration of US had a significant influence (*p* < 0.05) on the release of carbohydrates compared to the control group. The lowest contents were observed with MG for *S. coronopifolius* (60.43 mg/g DW) and TPP for *G. spinosum* (74.42 ± 6.47 mg/g DW). In light of these findings, US alone or in combination with other biomass pretreatment technique has enormous potential in intercellular carbohydrates release.

Strong ultrasonic power and long extraction time enhance cell wall disruption and consequently increase the total yield of glucose from macroalgae. This saccharidic fraction can constitute a very important fermentation substrate for heterotrophic microorganisms to produce biofuels.

### 3.7. Release of Pigments

**Figure 7:**
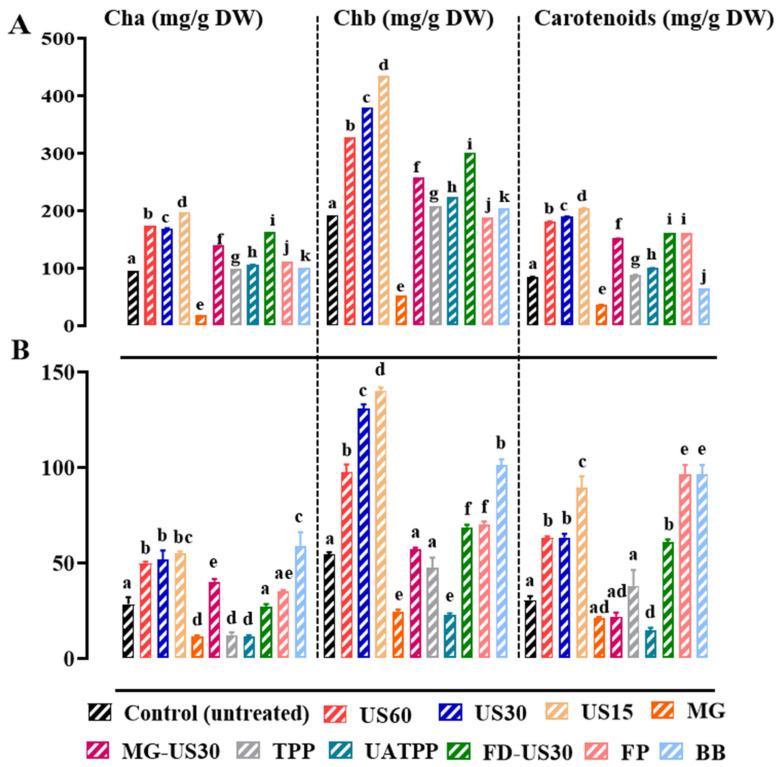
Pigment contents of *S. coronopifolius* (**A**) and *G. spinosum* (**B**) extracts using different techniques of cell disruption. a, b, c, d, e, f, g, h, i, j, and k: different letters mean significant differences between extracts of the same pigment (*p* < 0.05). Results are expressed as average ± standard deviation (SD) (*n* = 3). US, ultrasonication; MG, manual grinding; TPP, three-phase partitioning; UATPP, ultrasonication-assisted three-phase partitioning; MG-US30, manual grinding + ultrasonication for 30 min; FD-US30, freeze drying + ultrasonication for 30 min; FP, French press and BB, bead-beating.

*S. coronopifolius* and *G. spinosum* are photosynthetic algae rich in chlorophyll and carotenoids. Cell disruption, performed through grinding, homogenization, or ultrasonication, has been proven in previous research to significantly improve the effectiveness of pigment extraction [85,86]. In this study, the enhancement of pigment extraction using different pretreatment methods was assessed and the results are shown in Figure 7. The untreated cells and those pretreated with the MG method showed the lowest contents of chlorophyll a, b, and carotenoids. Regarding *S. coronopifolius*, the ultrasound pretreatment time (15 to 60 min) had a significant (*p* < 0.05) influence in the release of pigments. In fact, the highest contents were recorded for the US duration of 15 min with values of 197.94 mg/g DW, 435.34 mg/g DW, and 204 mg/g DW for chlorophyll a, chlorophyll b, and carotenoids, respectively.

Under the same conditions, the extracts of *G. spinosum* showed significant quantities of pigments estimated at 55.08 ± 0.92 mg/g DW, 140.29 ± 1.70 mg/g DW, and 89.33 ± 6.11 mg/g DW for chlorophyll a, chlorophyll b, and carotenoids, respectively. The pretreatment of this biomass by US for 30 min also released relatively considerable contents of chlorophyll a (51.93 ± 4.68 mg/g DW), chlorophyll b (130.93 ± 2.15 mg/g DW), and carotenoids (63.37 ± 2.00 mg/g DW).

Chlorophyll and carotenoids are located in chloroplasts and chromoplasts plastids, respectively, and the rupture of these organelles and the cell wall during US means a greater release of pigments. Temperature is an important parameter to be controlled since it directly impacts the solute and solvent properties. Therefore, the use of low or mild controlled temperatures is strongly recommended during the US process to avoid deterioration of thermolabile pigments.

It was shown that low-temperature sonication could improve cell rupture efficiency without negative mechanical or thermal impacts on sensitive carotenoids [65]. In a previous study, it was shown that cell disruption, achieved through grinding, homogenization, ultrasound or sonication, significantly improved the effectiveness of chlorophyll extraction using organic solvents [73].

### 3.8. Release of Phenolic Compounds

The quantification of TPC and TFC in the crude protein extracts (Table 1) shows that, except TPP, there is a significant difference (*p* < 0.05) between all pretreatments and untreated cells for both red macroalgae.

TPC values of the different protein extracts from *S. coronopifolius* and *G. spinosum* ranged from 4.85 ± 0.80 mg GAE/g DW to 21.00 ± 1.61 mg GAE/g DW and 14.66 ± 0.21 mg GAE/g DW to 32.79 ± 0.68 mg GAE/g DW, respectively. On the other hand, TFC values ranged from 0.70 ± 0.01 mg QE/g DW to 1.30 ± 0.11 mg QE/g DW and 0.38 ± 0.01 mg QE/g DW to 1.56 ± 0.05 mg QE/g DW for *S. coronopifolius* and *G. spinosum*, respectively. In addition, the maximum values of TPC and TFC for both red macroalgae were reached following US pretreatment for 60 min.

Among all the disruption techniques, the lowest TPC values were obtained by the TPP method which are 4.85 ± 0.80 GAE/g DW and 14.66 ± 0.21 mg GAE/g DW for *S. coronopifolius* and *G. spinosum*, respectively, while the lowest TFC levels were recorded after MG of *S. coronopifolius* (0.70 mg QE/g DW) and TPP of *G. spinosum* (0.48 ± 0.02 mg QE/g DW).

A comparison of the pretreatment techniques used showed that US for 60 min was superior in the release of phenolic compounds followed by the French press method. The extraction yield of polyphenols from natural bioresources is dependent on the solvent and method of extraction used. Water and aqueous mixtures of ethanol, methanol, and acetone are commonly used to extract plant materials [87]. Phenolics derived from red macroalgae are considered important components of both human and animal diets for their biological properties.

### 3.9. Antioxidant Activity

Due to the complexity of the oxidation process, the evaluation of the antioxidant capacity of molecules/products using a single test is insufficient to conclude their bioactivity with certainty. Therefore, the antioxidant activity of the crude protein extracts was measured using three in vitro antioxidant assays, including the DPPH free radical scavenging activity, reducing power, and ferrous ion-chelating ability assays [88].

**Figure 8:**
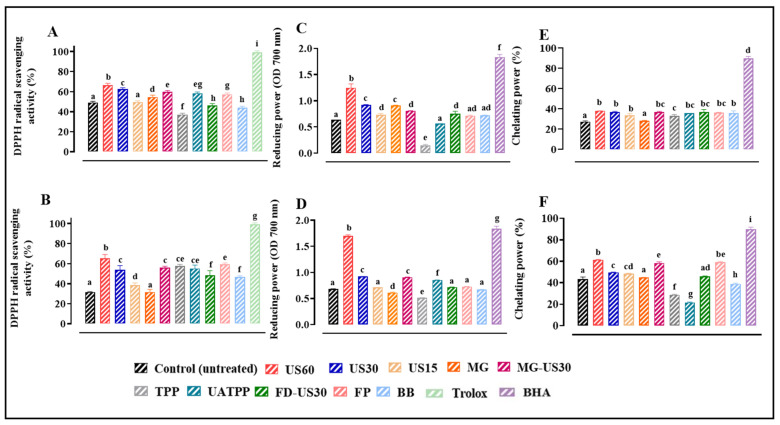
Antioxidant activity of *S. coronopifolius* and *G. spinosum* extracts using different techniques of cell disruption. DPPH free radical scavenging activity of *S. coronopifolius* (**A**) and *G. spinosum* (**B**); reducing power of *S. coronopifolius* (**C**) and *G. spinosum* (**D**); and ferrous ion-chelating ability of *S. coronopifolius* (**E**) and *G. spinosum* (**F**). a, b, c, d, e, f, g, h, and i: different letters mean significant differences between extracts of the same antioxidant test (*p* < 0.05). Results are expressed as average ± standard deviation (SD) (*n* = 3). US, ultrasonication; MG, manual grinding; TPP, three-phase partitioning; UATPP, ultrasonication-assisted three-phase partitioning; MG-US30, manual grinding + ultrasonication for 30 min; FD-US30, freeze drying + ultrasonication for 30 min; FP, French press and BB, bead-beating.

Free radical scavenging is a primary mechanism by which antioxidants inhibit oxidative processes. The DPPH radical scavenging assay is a commonly used method for evaluating the ability to scavenge free radicals generated by the DPPH reagent. As demonstrated in Figure 8A,B, the protein extracts derived from the two macroalgae biomasses pretreated with US for 60 min, 30 min, and 15 min showed significantly (*p* < 0.05) higher antioxidant activity compared to the untreated cells. In the case of the red algae *S. coronopifolius*, the highest DPPH scavenging activities were recorded for US 60 min (66.45 ± 1.84%) followed by US 30 min (62.43 ± 1.59%) and with the combined treatment MG-US30 (59.89 ± 1.31%) compared to the untreated cells (49.09 ± 1.35%). For the antioxidant potential of *G. spinosum* extracts, the most substantial DPPH scavenging activities were obtained with US for 60 min (65.16 ± 3.95%) followed by the French press method (59.42 ± 1.20%).

As mentioned before, the application of ultrasounds likely facilitated the release of bioactive compounds from red macroalgae, including, but not limited to, polyphenols, and as a consequence, extracts could exhibit a strong antioxidant capacity. Our results are in line with other research studies which showed that a significant DPPH radical scavenging activity was exerted by the protein extract (61.08%) of *Nanochloropsis* sp. prepared by ultrasound [73].

The reducing power assay is often used to evaluate the ability of natural antioxidants to donate an electron or hydrogen [89]. The reducing power of the protein extracts is shown in Figure 8C,D. Not surprisingly, US pretreatment presented the highest absorbances at 700 nm of 1.25 ± 0.07 and 1.70 ± 0.007 for *S. coronopifolius* and *G. spinosum*, respectively. The lowest reducing capacity was obtained with the TPP method for *S. coronopifolius* (0.145 ± 0.019) and *G. spinosum* (0.515 ± 0.001). Since the antioxidant activity of a constituent is directly related to its reducing power, this is a reliable method to evaluate the antioxidant capacities of various compounds [90]. Therefore, many reports have revealed that there is a direct correlation between antioxidant activities and the reducing power of certain bioactive compounds [91].

The chelation of Fe^2+^ was also used to determine the ability of protein extracts in metal-chelating activity. In the present study, the chelating activity of red macroalgae protein extracts, determined for each cell disruption technique, is shown in Figure 8E,F. The results revealed that the chelating power was significantly affected (*p* < 0.05) depending on the different biomass pretreatment methods applied. The percentage of the chelating ability of *S. coronopifolius* extracts varied from 27.46 ± 0.65% for untreated cells to 37.81 ± 0.25% for US with a duration of 60 min. However, this capacity was estimated to be 28.76 ± 0.45% and 61.37 ± 0.33% for the protein extracts of *G. spinosum* pretreated by TPP and US for 60 min, respectively. As in the two previous tests, the highest ferrous ion-chelating ability was obtained when US for 60 min was used for both red macroalgae. This potent antioxidant activity could be attributed to the TPC and TFC present in the protein extracts produced (Table 1).

Following the applied treatments, other non-protein components derived from macroalgae, such as polyphenols, carbohydrates, and pigments, were released simultaneously with the proteins. It is worth noting that red macroalgae possess unique biological characteristics due to their abundance in various bioactive compounds. These compounds contribute to the algae’s antioxidant, antibacterial, antiviral, and antifungal properties. Therefore, we conducted quantification and assessment of the biological activity of these co-released components to further elucidate their functional properties.

Indeed, it has been reported that phenolic compounds are a significant source of natural antioxidants and have been demonstrated to be more potent antioxidants than vitamins C, E, and carotenoids [92]. Siriwardhana et al. [93] reported that components such as low molecular weight polysaccharides, pigments, proteins, and peptides also influence the antioxidant activity of the extract. Red algae are known to contain phenolic acids such as gallic acid, gentisic acid, and protocatechuic acid [94], which contribute to the rich phytochemical composition of these bioresources and consequently could have shown greater antioxidant activity. Furthermore, flavonoids compounds were known to also possess antioxidant activities [95,96].

In addition, pigments like carotenoids contribute significantly to the antioxidant activity of macroalgae extracts. Carotenoids are considered antioxidants because of their ability to deactivate and trap free radical, especially singlet oxygen quenching [97,98,99]. A lot of research has proven the antioxidant properties of algal carotenoids and the role that they play in preventing many diseases associated with oxidative stress [100]. Furthermore, the presence of these abundant polyphenols suggests evidence on behalf of macroalgae as a potential source of antioxidants for application in the functional foods, packaging, cosmetics, and pharmaceutical industries, while further toxicity, animal, and clinical studies may be required for human use [101].

## 4. Conclusions

This study highlights the potential of eight different pretreatments to enhance the release of bioactive compounds, particularly proteins, from two red macroalgae *S. coronopifolius* and *G. spinosum*. Among all the tested techniques, ultrasonication for 1 h at 20 kHz, 200 W and 90% of amplitude could be a promising method to improve cell disruption not only for proteins but also to recover a wider range of high-value compounds such as phenolic compounds, flavonoids, and carbohydrates. The analysis of protein extracts obtained with ultrasonication pretreatment using SDS-PAGE showed a broad spectrum of bands. In addition, the antioxidant analysis revealed high antioxidant activity, including free radical scavenging activity, reducing power, and iron-chelating activity. This potent antioxidant activity could be attributed to the polyphenols and flavonoids present in the protein extracts produced. Overall, the study highlights the potential of ultrasonication treatment in efficiently disrupting the cell walls of macroalgae and extracting important molecules of interest, especially proteins, thus offering promising opportunities for further research and application in various fields.

## Figures and Tables

**Table 1:** Total polyphenol content and flavonoid content in *S. coronopifolius* and *G. spinosum* extracts using different techniques of cell disruption. Results are expressed as average ± standard deviation (SD) (*n* = 3). a, b, c, d, e, and f: different letters mean significant differences between extracts for the same analysis (*p* < 0.05). US, ultrasonication; MG, manual grinding; TPP, three-phase partitioning; UATPP, ultrasonication-assisted three-phase partitioning; MG-US30, manual grinding + ultrasonication for 30 min; FD-US30, freeze drying + ultrasonication for 30 min; FP, French press and BB, bead-beating.

	*S. coronopifolius*		*G. spinosum*	
	TPC (mg GAE/g of DW)	TFC (mg QE/g of DW)	TPC (mg GAE/g of DW)	TFC (mg QE/g of DW)
Control (untreated)	5.35 ± 0.30 ^a^	0.71 ± 0.09 ^a^	24.62 ± 0.36 ^a^	0.38 ± 0.01 ^a^
US 60 min	21.00 ± 1.61 ^b^	1.30 ± 1.11 ^b^	32.79 ± 0.68 ^b^	1.56 ± 0.05 ^b^
US 30 min	9.78 ± 1.11 ^c^	1.23 ± 1.97 ^b^	28.02 ± 0.74 ^c^	1.00 ± 0.02 ^c^
US 15 min	8.35 ± 0.70 ^d^	0.98 ± 0.01 ^c^	25.61 ± 0.21 ^ac^	0.73 ± 0.02 ^d^
MG	7.21 ± 0.10 ^d^	0.70 ± 0.09 ^a^	22.79 ± 2.28 ^a^	0.69 ± 0.03 ^d^
MG-US30	9.50 ± 1.31 ^cd^	1.03 ± 0.47 ^cd^	30.57 ± 1.83 ^bc^	0.95 ± 0.02 ^e^
TPP	4.85 ± 0.80 ^a^	0.84 ± 0.62 ^e^	14.66 ± 0.21 ^d^	0.48 ± 0.02 ^e^
UATPP	11.14 ± 0.00 ^e^	0.88 ± 0.41 ^e^	16.68 ± 3.02 ^d^	1.03 ± 0.02 ^c^
FD-US30	10.35 ± 0.30 ^ce^	1.01 ± 1.03 ^cd^	28.66 ± 0.21 ^c^	1.00 ± 0.07 ^ce^
FP	9.14 ± 0.20 ^cd^	1.06 ± 0.60 ^d^	32.42 ± 1.04 ^b^	0.86 ± 0.02 ^f^
BB	7.57 ± 0.20 ^d^	0.35 ± 0.64 ^f^	22.03 ± 2.13 ^a^	0.69 ± 0.02 ^d^

## Data Availability

The original contributions presented in the study are included in the article, further inquiries can be directed to the corresponding author.
